# Harnessing a Lytic (*Caudoviricetes* with Podovirus-Like Morphology) Bacteriophage (ØAS2) for Biocontrol of Multidrug-Resistant *Serratia marcescens* Biofilms in Milk and Soft Cheese

**DOI:** 10.3390/biology15131055

**Published:** 2026-07-02

**Authors:** Dalia Kamal Rawy, Fawziah M. Albarakaty, Rehab M. A. El-Desoukey, Mayasar I. Al-Zaban, Alya Aljuaid, Mohammed Aladhadh, Khalid A. Alsaleem, Raghda M. S. Moawad

**Affiliations:** 1 Student Hospital, Assiut University Hospitals, Assiut University, Assiut 71515, Egypt; dalia.rawy@aun.edu.eg; 2 Department of Biology, Faculty of Science, Umm Al-Qura University, P.O. Box 715, Makkah 21955, Saudi Arabia; fmbarakati@uqu.edu.sa; 3 Microbiology and Immunology Department, National Research Centre, Giza 12622, Egypt; 4 Applied College Afif, Shaqra University, Shaqra 11961, Saudi Arabia; 5 Department of Biology, College of Science, Princess Nourah Bint Abdulrahman University, P.O. Box 84428, Riyadh 11671, Saudi Arabia; mialzaban@pnu.edu.sa; 6 Department of Biology, College of Science and Humanities, Shaqra University, Shaqra 11961, Saudi Arabia; aaljuaied@su.edu.sa; 7 Department of Food Science and Human Nutrition, College of Agriculture and Food, Qassim University, Buraydah 51452, Saudi Arabia; aladhadh@qu.edu.sa (M.A.); k.alsaleem@qu.edu.sa (K.A.A.); 8 Dairy Department, Faculty of Agriculture, Minia University, Minia 61519, Egypt; raghda.moawad@mu.edu.eg

**Keywords:** bacteriophages, antibiotic resistance, multidrug-resistant bacteria, biofilm eradication, dairy spoilage, *Caudoviricetes*, food bio preservation, lytic activity

## Abstract

*Serratia marcescens* is a nosocomial pathogen that has acquired resistance to multiple antibiotics, necessitating alternative antimicrobial strategies. The aim of this study was to isolate and characterize a novel phage (ØAS2) against *Serratia marcescens* and evaluate its biocontrol potential in dairy matrices. In this study, a lytic bacteriophage specific to *S. marcescens*, designated ØAS2, was isolated from sewage samples collected in Assiut, Egypt. Phage ØAS2 was characterized using plaque assays, transmission electron microscopy (TEM), host range determination, pH and thermal stability tests, and one-step growth curve analysis. Its ability to inhibit bacterial growth and disrupt biofilms was also evaluated in vitro. TEM revealed that ØAS2 possesses an icosahedral head approximately 47.2 nm in diameter and a very short tail, consistent with the morphology of a member of the class *Caudoviricetes* that exhibits podovirus-like morphology. The phage exhibited a broad host range, infecting various *Serratia* strains as well as other Gram-negative bacteria, including *Klebsiella* spp., *Escherichia coli*, *Salmonella typhi*, and *Shigella* spp. ØAS2 was thermostable up to 60 °C and showed maximum activity at pH 8. One-step growth curve analysis revealed a short latent period of 10 min and a burst size of 115 PFU per infected cell. ØAS2 effectively inhibited the growth of *S. marcescens* SM02 in vitro and significantly reduced preformed biofilms at different multiplicities of infection (MOIs). When applied to skim milk and fresh soft cheese at various MOIs (Multiplicities of Infection), the phage successfully controlled bacterial contamination under refrigerated storage (7 °C for 7–10 days). At MOI 5.0, phage ØAS2 reduced biofilm biomass by 25.6%, planktonic growth by 85.7%, and achieved a reduction of 2.1 log_10_ CFU/mL in skim milk. These findings indicate that ØAS2 is a promising biocontrol candidate for managing *S. marcescens* spoilage in dairy products.

## Simple Summary

*Serratia marcescens* is a harmful bacterium that can contaminate milk and dairy products, causing spoilage and posing health risks due to its resistance to multiple antibiotics. In this study, we isolated a natural virus called bacteriophage ØAS2 from sewage water in Assiut, Egypt, which specifically kills this bacterium. This phage has a very short tail (as a member of the class *Caudoviricetes* that exhibits *podovirus*-like morphology) and works quickly, with a latent period of only 10 min and a burst size of 115 new phages per infected bacterial cell. It remains stable at temperatures up to 60 °C and across a wide pH range (4–11), making it suitable for dairy environments. Importantly, ØAS2 not only stops bacterial growth but also destroys the biofilms that protect the bacteria. When applied to skim milk and fresh soft cheese under refrigeration, the phage successfully reduced *S. marcescens* counts. These findings introduce a new, potentially safe, and eco-friendly biocontrol tool that can help reduce spoilage and extend the shelf life of dairy products without using chemical preservatives or antibiotics.

## 1. Introduction

The rapid emergence and global spread of antimicrobial resistance (AMR) represent one of the most serious challenges to global public health. The increasing prevalence of multidrug-resistant (MDR) bacteria has compromised the effectiveness of conventional antimicrobial therapies, resulting in prolonged infections, increased healthcare costs, and elevated mortality rates. Consequently, there is an urgent need for innovative and sustainable antimicrobial strategies capable of controlling MDR pathogens while reducing dependence on traditional antibiotics [1].

Among MDR bacterial pathogens, *Serratia marcescens* is an opportunistic Gram-negative bacterium belonging to the family *Enterobacteriaceae*. It is frequently associated with nosocomial infections, including urinary tract infections, wound infections, respiratory tract infections, and bloodstream infections, particularly among immunocompromised patients [2,3]. In addition to its clinical importance, *S. marcescens* is widely distributed in environmental reservoirs and food-processing environments. The remarkable adaptability of this organism, combined with its intrinsic and acquired resistance mechanisms, contributes to its persistence in healthcare and industrial settings, making it difficult to control using conventional antimicrobial approaches [1,3].

*S. marcescens* can survive in diverse habitats, including water, soil, plants, animals, and food-processing environments [3]. One of its most important virulence and persistence factors is its ability to form biofilms, which are structured microbial communities embedded within a self-produced extracellular polymeric substance (EPS) matrix [4,5]. Biofilm formation enhances bacterial adhesion to surfaces and protects against environmental stresses, disinfectants, and antimicrobial agents. Consequently, biofilm-associated cells exhibit increased tolerance to antimicrobial treatments and contribute significantly to the persistence and dissemination of MDR bacteria [4,5,6].

In the dairy industry, *S. marcescens* has emerged as an important spoilage-associated microorganism because of its ability to survive under refrigerated storage conditions and produce extracellular proteolytic and lipolytic enzymes. These enzymes degrade milk proteins and fats, leading to undesirable sensory defects such as bitterness, off-flavors, discoloration, and reduced product shelf life. Furthermore, the ability of *S. marcescens* to establish biofilms on dairy-processing surfaces facilitates long-term persistence within production facilities and increases the risk of recurrent product contamination. The occurrence of MDR *S. marcescens* strains in dairy environments further emphasizes the need for effective alternative control strategies capable of ensuring food safety and product quality [4,5,6].

Given the increasing challenges associated with MDR bacteria and biofilm-mediated contamination, bacteriophages have gained considerable attention as promising biological control agents. Phages are viruses that specifically infect bacterial hosts and are considered the most abundant biological entities on Earth. Owing to their high host specificity, self-replicating nature, and ability to target antibiotic-resistant bacteria, phages represent an attractive alternative or complementary approach to conventional antimicrobial interventions [7,8,9].

Bacteriophages are highly diverse and employ sophisticated infection mechanisms to replicate within susceptible bacterial hosts [10,11]. Phage infection begins with adsorption to specific bacterial receptors, including lipopolysaccharides, membrane proteins, capsules, and other surface-associated structures [12]. Following adsorption, the phage injects its genetic material into the host cell and hijacks the bacterial metabolic machinery to produce progeny virions [11]. The subsequent action of phage-encoded lytic proteins results in bacterial cell lysis and release of newly formed phage particles capable of infecting neighboring cells [11]. In addition, the combined use of bacteriophages and antibiotics has shown promising potential for enhancing antimicrobial efficacy and limiting the development of bacterial resistance [9].

Phages also offer several advantages for food applications. Their high specificity enables selective targeting of undesirable bacteria while preserving beneficial microbiota, thereby minimizing microbiome disruption. Bacteriophages exhibit remarkable host selectivity, allowing them to eliminate specific bacterial pathogens without adversely affecting beneficial microbial communities. This characteristic represents a major advantage of phage-based biocontrol over broad-spectrum antimicrobial agents. Moreover, bacteriophages have demonstrated considerable potential for controlling foodborne pathogens and biofilm-forming bacteria in food-processing environments, contributing to improved food safety and product quality without adversely affecting the organoleptic characteristics of foods [13,14].

Despite the growing interest in phage-based biocontrol, limited information is available regarding the application of lytic bacteriophages against multidrug-resistant *S. marcescens* and its biofilms in dairy products. Furthermore, studies evaluating the efficacy of *Serratia*-specific phages in milk and soft cheese remain scarce [13,14]. Therefore, the present study aimed to isolate and characterize a novel lytic bacteriophage (ØAS2) targeting multidrug-resistant *S. marcescens* and to evaluate its potential as a biocontrol agent for reducing bacterial growth and biofilm formation in dairy matrices.

## 2. Materials and Methods

### 2.1. Bacterial Strains and Growth Conditions

*S. marcescens* isolates were obtained from sewage water samples collected from multiple locations in Assiut, Egypt. Approximately 500 mL of wastewater was collected from municipal wastewater treatment plants in March 2024. Samples were transported under sterile conditions and processed within 6–7 h of collection. Each sample was processed individually without pooling. For bacterial isolation, 1 mL of each wastewater sample was diluted in 9 mL of sterile distilled water and homogenized by shaking for 20 min. Serial dilutions were prepared, and aliquots were plated on MacConkey agar (Oxoid, United Kingdom). Plates were incubated at 37 °C for 24 h. isolated colonies were purified and maintained under appropriate conditions for subsequent identification. All experiments were conducted in three independent biological replicates, each comprising three technical replicates (n = 3).

### 2.2. Identification of Serratia Isolate

The isolate was identified as *S. marcescens* using the VITEK 2 C1 system (version 04.01, BioMérieux, France) at the Clinical Pathology Laboratory of Assiut University Hospital, Assiut, Egypt. For accurate identification, 16S rRNA sequencing was performed using the Ready Reaction BigDye Terminator v3.1 Cycle Sequencing Kit (Perkin-Elmer/Applied Biosystems, Foster City, CA, USA; Cat. No. 4336817, Sigma, Giza, Egypt). The obtained 16S rRNA sequence was deposited in the GenBank database under accession number PZ282064.1. The isolated strain was stored under appropriate conditions for subsequent experiments.

### 2.3. Antibiotic Susceptibility Testing

Antibiotic susceptibility was determined using the Kirby–Bauer disk diffusion method according to Clinical and Laboratory Standards Institute (CLSI) guidelines [15]. *S. marcescens* SM02 was tested against 12 antibiotic disks: amikacin (AK, 30 μg), imipenem (IPM, 10 μg), lincomycin (MY, 10 μg), penicillin (P, 10 μg), amoxycillin (AML, 10 μg), erythromycin (E, 15 μg), kanamycin (K, 30 μg), azithromycin (AZM, 15 μg), moxifloxacin (5 μg), cefepime (FEP, 30 μg), ofloxacin (OFX, 5 μg), and vancomycin (VA, 30 μg) (all from Oxoid, England). Disks were placed on Mueller–Hinton agar (MHA) plates, and after overnight incubation at 37 °C, inhibition zone diameters were measured [16]. Strain SM02 was selected for further characterization and biocontrol experiments because it exhibited multidrug resistance and demonstrated high susceptibility to phage ØAS2, making it an appropriate representative host.

### 2.4. Isolation, Propagation, and Purification of Bacteriophage

A lytic phage against *S. marcescens* SM02 was isolated from sewage water samples from Assiut, Egypt, following an enrichment protocol [17]. Sewage samples were clarified by centrifugation at 6000× *g* for 20 min and filtered through a 0.45 μm membrane filter. The supernatant was filtered through a 0.45 μm membrane filter to remove bacterial cells while allowing phage particles (typically <0.1 μm) to pass through. This approach is widely used in phage isolation protocols to obtain bacteria-free lysates without compromising phage recovery efficiency. For enrichment, 100 mL of wastewater was mixed with 100 mL of nutrient broth and 5 mL of overnight bacterial culture (10^6^ CFU/mL) and incubated at 37 °C for 24 h with shaking at 120× *g*. After incubation, the culture was centrifuged at 6000× *g* for 10 min at 4 °C, and the supernatant was filtered sequentially through 0.45 μm membrane filters (Millex™ sterile syringe filters). Phage concentration (PFU/mL) was determined by agar overlay assay. The phage was propagated and purified by three successive single-plaque isolations, as described previously [18]. Phage concentration (PFU/mL) was determined using the double-layer agar overlay assay. Briefly, 100 µL of phage suspension was mixed with 200 µL of host bacterial culture and 3 mL of molten soft agar (0.7%), then immediately poured onto nutrient agar plates. After solidification, plates were incubated at 37 °C overnight, and plaques were counted to determine phage titers. A single plaque was picked and transferred to 3.0 mL of nutrient broth (Oxoid Ltd., Basingstoke, UK; Cat. No. CM0001B) containing 100 μL of the bacterial host, then incubated at 37 °C with shaking at 120× *g*. After incubation, the mixture was centrifuged at 6000× *g* for 10 min, and the supernatant was filtered through a 0.45 μm sterile filter. Following filtration through a 0.45 μm membrane, the phage suspension was subjected to repeated rounds of plaque purification. In addition, chloroform treatment was applied during phage preparation to minimize the possibility of bacterial contamination. The purified phage was stored at 4 °C.

### 2.5. Morphological Characterization by Electron Microscopy

Purified *Serratia* phage was stained with sodium phosphotungstate and examined by transmission electron microscopy (TEM) according to Didamony et al. [19]. A drop of phage suspension (10^8^ PFU/mL) was placed on 200-mesh copper grids coated with carbon-coated Formvar films. Excess liquid was removed with filter paper. A saturated solution of sodium phosphotungstate was then applied, and any excess was removed. Specimens were examined using a JEOL JEM-1400 TEM at 80 kV (Electron Microscope Unit, Faculty of Agriculture, Mansoura University, Egypt).

### 2.6. Host Range Determination

The host range of ØAS2 was evaluated against various *S. marcescens* isolates and other bacterial strains, including *Klebsiella* spp., *Escherichia coli*, *Salmonella typhi*, and *Shigella* spp., that were obtained from the Faculty of Agriculture, Minia University, Egypt. Each bacterial strain was prepared on nutrient agar plates. A 10 μL drop of the isolated phage was placed on each bacterial lawn and incubated at 37 °C for 24 h. Plaque formation was observed as an indicator of lytic activity [20].

### 2.7. Phage Adsorption Rate

Phage adsorption was measured as described by Jamal et al. [21]. Phage suspension was added to *S. marcescens* cultures (0.6 × 10^8^ CFU/mL) at an MOI of 0.1 and incubated at 37 °C with shaking. Samples were collected at 5 min intervals for 30 min. Chloroform (two drops) was added to each sample to release adsorbed phages, and free phage particles were quantified using the double-layer agar method. The initial phage titer at time zero (T0) was used as the baseline value. A reduction in free phage titer indicated successful adsorption to host cells. The adsorption rate (%) was calculated as follows: [(initial PFU–unadsorbed PFU)/initial PFU] × 100.

### 2.8. One-Step Growth Curve

One-step growth curve analysis was performed according to Pajunen et al. [22]. Phage suspension was added to *S. marcescens* cultures (0.6 × 10^8^ CFU/mL) at an MOI of 0.1 and incubated at 37 °C for 10 min to allow for adsorption. The mixture was centrifuged at 10,000× *g* for 10 min to remove unadsorbed phage particles. The pellet was then resuspended in 10 mL of nutrient broth and incubated at 37 °C. Samples were collected at 10 min intervals for 80 min, and phage titers were determined using the double-layer agar method. The initial phage titer at time zero (T0) was measured immediately after adsorption and used as the baseline. The burst size was calculated using the following equation: (final PFU after lysis–initial PFU) divided by the number of infected bacterial cells.

### 2.9. Thermal and pH Stability

Thermal stability was assessed as described by Mahmoud et al. [18]. Phage suspension (10^6^–10^8^ PFU/mL) was incubated at different temperatures (30, 40, 50, 60, 70, 80, 90, and 100 °C) for 10 min in a water bath. After incubation, phage infectivity was assayed using the double-layer agar plate method. For pH stability analysis, aliquots of phage suspension (10^6^–10^8^ PFU/mL) were added to nutrient broth adjusted to pH values ranging from 3.0 to 12.0. To ensure accurate pH control, the following buffer systems were used: citrate–phosphate buffer (pH 3–7), Tris–HCl buffer (pH 8–9), and carbonate–bicarbonate buffer (pH 10–11). Samples were incubated at 4 °C overnight, and phage viability was determined using the double-layer agar overlay method [23].

### 2.10. Lytic Activity of ØAS2 Against S. marcescens

The antibacterial activity of ØAS2 against *S. marcescens* SM02 was determined at MOIs of 0.1, 1.0, and 5.0. SM02 was grown in 10 mL of nutrient broth to an OD_600_ of 0.2 (approximately 0.6 × 10^8^ CFU/mL). ØAS2 phage was added at the indicated MOIs and incubated at 37 °C. Bacterial growth was monitored by measuring OD_600_ every 4 h for 24 h using a UV spectrophotometer (UNICO 2100, United Products & Instruments, Dayton, NJ, USA). Experiments were performed in triplicate [17]. The following bacterial strains were used in these experiments: *S. marcescens* SM02 PZ282064.1 (main host), *Serratia marcescens* SM01 PZ273769.1, *Serratia marcescens* SM03, *Serratia marcescens* SM04, *Serratia marcescens* SM05, *E. coli* ATCC 25922, *E. coli* M30LC649234.1, *Salmonella typhi* 1, *Salmonella typhi* ATCC14028, *Shigella* spp., *Klebsiella oxytoca* KO37, *K. pneumoniae* KP77 OK326738.1, *K. pneumoniae* KP98, *K. pneumoniae* KP75 OK326736.1 and *K. pneumoniae* KP72 OK326732.1. Optical density (OD600) measurements were used as a rapid and widely accepted preliminary indicator of bacterial growth suppression in phage-bacteria interaction studies. We acknowledge that viable cell enumeration would provide a more direct assessment of bacterial reduction.

### 2.11. Biofilm Eradication Assay

The ability of ØAS2 to eradicate preformed *S. marcescens* biofilms was assessed according to Taha et al. [24]. Biofilms were developed by adding 200 μL of bacterial culture (10^8^ CFU/mL) to each well of a 96-well flat-bottom polystyrene microtiter plate and incubating at 37 °C for 24 h with gentle shaking. After biofilm formation, the supernatant was discarded, and wells were washed twice with 0.9% NaCl to remove unattached planktonic cells. Plates were dried at 37 °C for 1 h. Phage ØAS2 diluted in 0.9% NaCl was added to wells at MOIs of 0.1, 1.0, and 5.0; control wells received sterile saline. Plates were incubated at 37 °C with shaking for 24 h. After incubation, contents were discarded, wells were washed as before, and plates were dried at 37 °C for 1 h. Total biofilm biomass was determined by staining with 1% crystal violet for 20 min. Plates were washed with distilled water and air-dried. The bound dye was solubilized by adding 200 μL of 95% ethanol to each well, and absorbance was measured at OD_570_ using a microplate reader. All assays were performed as three independent experiments (n = 3).

### 2.12. Phage Activity in Skim Milk

The bacteriostatic effect of ØAS2 against *S. marcescens* SM02 in skim milk at 7 °C was evaluated following Zhang et al. [25] with modifications. Briefly, 100 μL of overnight culture of *S. marcescens* SM02 was added to 10 mL of skim milk to obtain a final concentration of 10^4^ CFU/mL. Then, 100 μL of phage suspension at varying concentrations (10^6^–10^8^ PFU/mL) was added to achieve MOIs of 0, 0.1, 1.0, and 5.0. Samples were incubated at 7 °C for 7 days. *S. marcescens* counts were determined using the plate-counting method [26].

### 2.13. Cheese Manufacturing and Phage Application

Soft cheese was manufactured according to Moneeb et al. [27] at the Dairy Products Laboratory, Faculty of Agriculture, Minia University, Egypt. Control cheese samples were prepared, then artificially contaminated with *S. marcescens* SM02 and kept at refrigerated temperatures for 30 min. The initial contamination level of *S. marcescens* SM02 in cheese samples was 10^4^ CFU/g before phage application. Phage suspension was added at MOIs of 0, 3, and 5. *S. marcescens* SM02 was enumerated over 10 days of refrigerated storage.

### 2.14. Statistical Analysis

Data are expressed as mean ± standard deviation (SD) from three independent experiments (n = 3). Statistical analyses were performed using one-way analysis of variance (ANOVA) with CoStat software (version 6.4, CoHort Software, Monterey, CA, USA) [28]. Differences were considered statistically significant if *p* ≤ 0.05.

## 3. Results

### 3.1. Antibiotic Resistance Profile

*S. marcescens* SM02 was evaluated for susceptibility to 12 antimicrobial agents (Table 1). Based on CLSI-interpretable antibiotics, the isolate exhibited high susceptibility to ofloxacin, cefepime, moxifloxacin, imipenem, and azithromycin, while showing intermediate susceptibility to kanamycin. Resistance was observed against amikacin and amoxicillin. Several antimicrobial agents, including lincomycin, erythromycin, penicillin, and vancomycin, were not assigned CLSI susceptibility categories because interpretive criteria for Serratia marcescens are not available. The observed resistance profile to multiple antimicrobial classes supports the classification of strain SM02 as a multidrug-resistant isolate. Ofloxacin and moxifloxacin belong to the fluoroquinolone class, cefepime is a fourth-generation cephalosporin, and imipenem is a carbapenem antibiotic.

### 3.2. Morphological Characterization of Phage ØAS2

**Figure 1:**
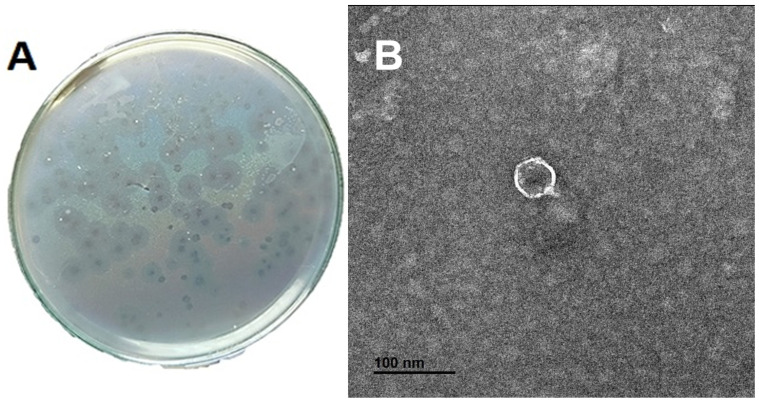
Morphological characterization of phage ØAS2. (**A**) Clear plaques formed on *S. marcescens* SM02 lawn, indicating lytic activity. (**B**) Transmission electron micrograph of phage ØAS2 showing an icosahedral head and a very short tail (scale bar = 100 nm; magnification = 40,000×).

ØAS2 formed clear, regular plaques with a dark center and a translucent halo on the SM02 bacterial lawn, with an average diameter of 4.36 ± 0.61 mm (Figure 1A). TEM imaging revealed an icosahedral head with an average diameter of 47.28 ± 2.93 nm and a very short tail of approximately 7.93 ± 2.35 nm (Figure 1B), consistent with the morphology of a member of the class *Caudoviricetes* that exhibits podovirus-like morphology.

### 3.3. Host Range

ØAS2 demonstrated lytic activity against several *Serratia* strains (SM02, *Serratia marcescens* 1, *Serratia marcescens* 2, *Serratia marcescens* 3, and *Serratia marcescens* 4), with three strains being susceptible (Table 2). Although ØAS2 infected 3 of 4 tested *S. marcescens* strains, a larger collection (10–15 isolates or more) would be required for a comprehensive host-range evaluation. Among other Gram-negative bacteria, ØAS2 infected *E. coli* ATCC 25922, *Salmonella typhi* ATCC14028, and *Shigella* spp., as well as *Klebsiella oxytoca* KO37 and *Klebsiella pneumoniae* KP77 OK326738.1. In contrast, *K. pneumoniae* KP98, KP75 OK326736.1, and KP72 OK326732.1 were resistant. These results suggest a relatively broad host range for OAS2 among the bacterial isolates tested.

### 3.4. Phage Adsorption Rate

**Figure 2:**
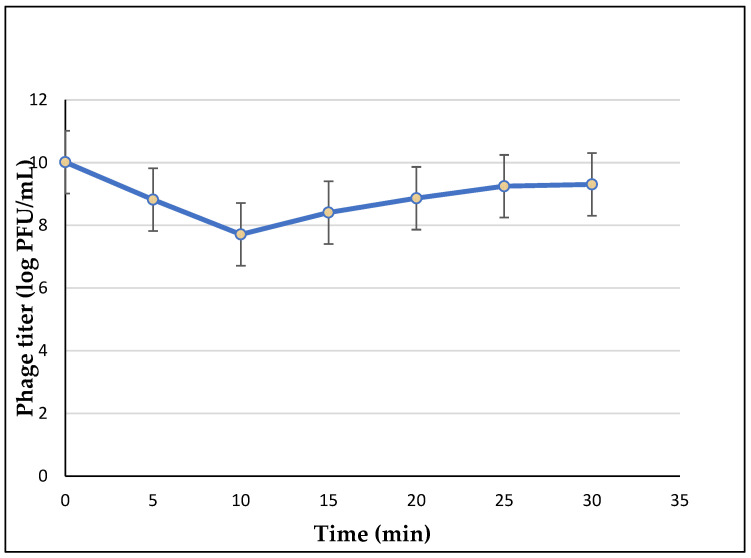
Adsorption rate of phage ØAS2 at MOI = 0.1. Data are presented as mean ± standard deviation (SD) from three independent experiments (n = 3). Error bars represent SD. *p* ≤ 0.05.

At an MOI of 0.1, phage ØAS2 adsorbed rapidly to bacterial cells. Maximum adsorption (99.51%) was achieved after 10 min of incubation (Figure 2) (Adsorption rate (%) = [(PFU at T_0_ − PFU at T_3_)/PFU at T_0_] × 100; PFU at T_0_ = 1.04 × 1010; PFU at T_X_ = 5.10 × 107; calculated Adsorption rate = 99.51%).

### 3.5. One-Step Growth Curve

**Figure 3:**
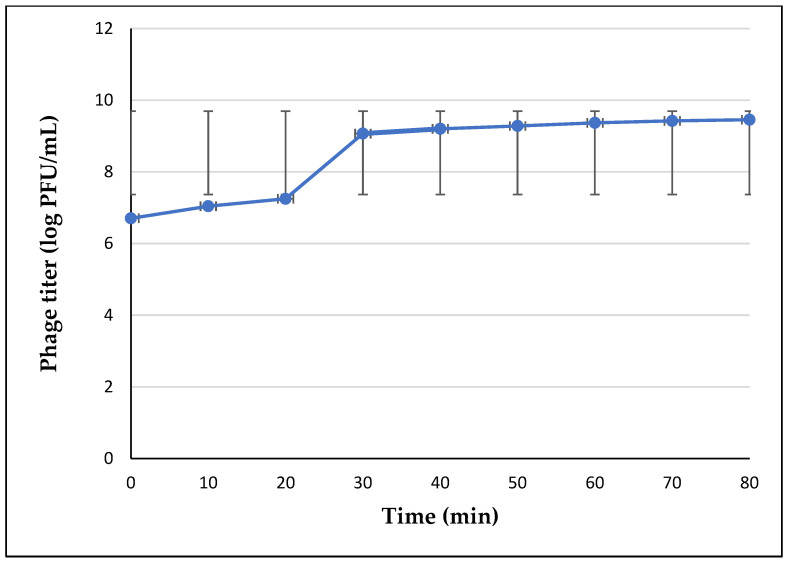
One-step growth curve of phage ØAS2. All data are presented as mean ± standard deviation (SD) from three independent experiments (n = 3). Error bars represent SD.

The one-step growth curve (Figure 3) showed that ØAS2 had a latent period of 10 min, followed by a rise period of 20 min. This pattern is clearly illustrated in Figure 3, where an initial latent phase is followed by a sharp increase in phage titer and a subsequent plateau phase. The complete infection cycle from adsorption to progeny release lasted approximately 30 min. The burst size was calculated to be 115 PFU per infected cell. According to the formula, Burst size = (final phage titer − initial phage titer after adsorption)/initial bacterial count, the burst-size according to raw experimental values is ~115 PFU/cell (Initial bacterial count = 2 × 10^8^ CFU/mL; phage count after adsorption = 5.2 × 10^7^; PFU/mL; plateau phage count = 2.9 × 10^10^ PFU/mL).

### 3.6. Thermal and pH Stability

**Figure 4:**
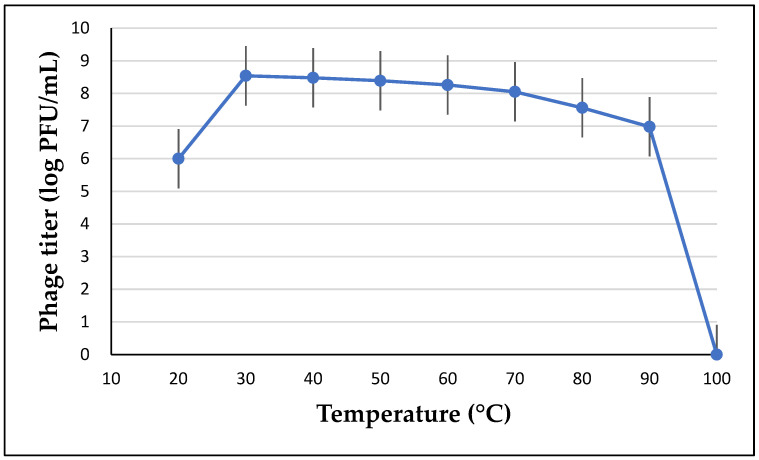
Thermal stability of phage ØAS2 (10 min exposure at indicated temperatures). All data are presented as mean ± standard deviation (SD) from three independent experiments (n = 3). Error bars represent SD.

**Figure 5:**
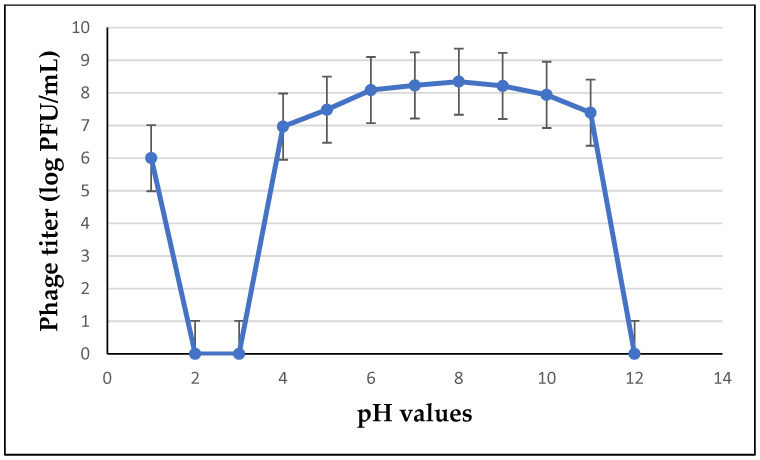
pH stability of phage ØAS2 (overnight incubation at 4 °C). All data are presented as mean ± standard deviation (SD) from three independent experiments (n = 3). Error bars represent SD.

ØAS2 remained stable up to 60 °C, with infectivity retained after 10 min of exposure, indicating moderate thermostability (Figure 4). At 70 °C and above, phage activity was drastically reduced. Regarding pH stability, ØAS2 maintained activity across a broad range (pH 4–11), with optimal activity at pH 8 (Figure 5).

### 3.7. Lytic Activity of ØAS2 In Vitro

**Figure 6:**
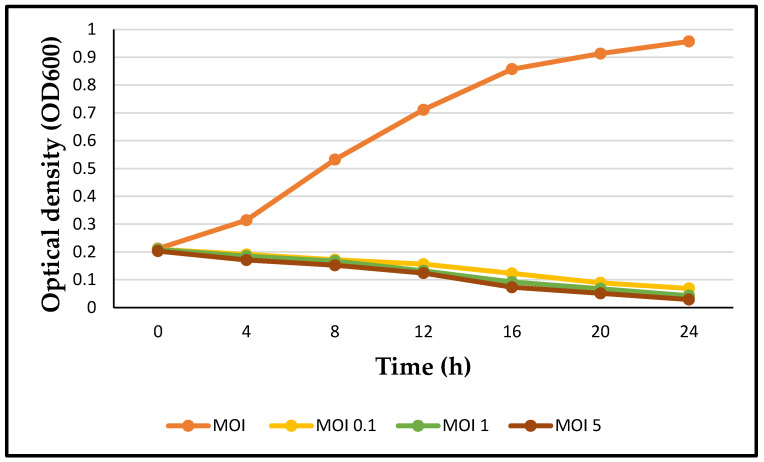
Lytic activity of phage ØAS2 against *S. marcescens* SM02 at different multiplicities of infection (MOIs). Optical density (OD600) was monitored over 24 h. Data are presented as mean ± standard deviation (SD) from three independent experiments (n = 3). Error bars represent SD.

Phage ØAS2 significantly inhibited the growth of *S. marcescens* SM02 at all tested multiplicities of infection (MOIs: 0.1, 1.0, and 5.0) compared to the untreated control over 24 h (Figure 6). The inhibitory effect was clearly dose-dependent, with MOI 5.0 exhibiting the most pronounced reduction in bacterial growth.

### 3.8. Biofilm Eradication

**Figure 7:**
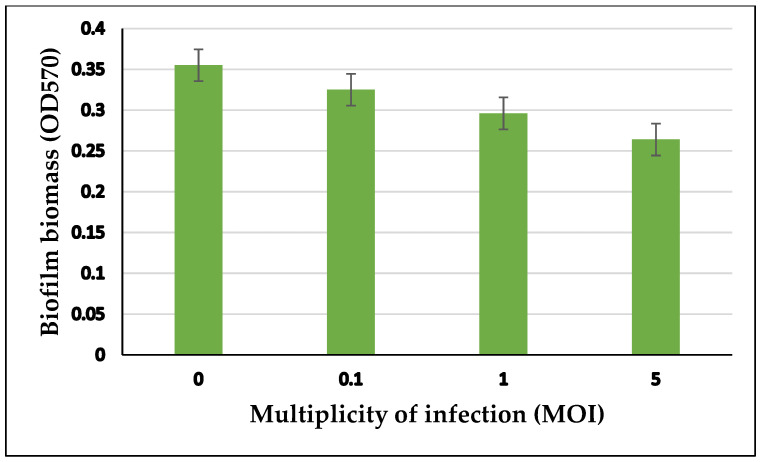
Effect of phage ØAS2 at different multiplicities of infection (MOIs) on preformed *S. marcescens* SM02 biofilms. Biofilm biomass (OD570) was measured as an indicator of biofilm formation. Data are presented as mean ± standard deviation (SD) from three independent experiments (n = 3). Error bars represent SD.

Treatment with phage ØAS2 significantly reduced preformed *S. marcescens* biofilms’ biomass after 24 h of exposure. Biofilm reduction was observed at all tested multiplicities of infection (MOIs: 0.1, 1.0, and 5.0), with the greatest reduction achieved at MOI 5.0, indicating a dose-dependent antibiofilm effect (Figure 7).

### 3.9. Inhibition of S. marcescens in Skim Milk

**Figure 8:**
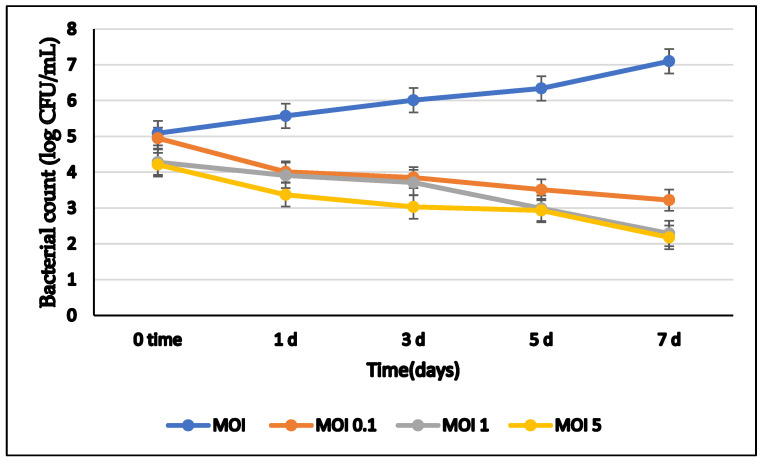
Effect of phage ØAS2 at different multiplicities of infection (MOIs) on *S. marcescens* SM02 counts in skim milk during storage at 7 °C for 7 days. Bacterial counts are expressed as log CFU/mL. Data are presented as mean ± standard deviation (SD) from three independent experiments (n = 3). Error bars represent SD.

In skim milk stored at 7 °C for 7 days, phage ØAS2 effectively inhibited the growth of *S. marcescens* SM02 (Figure 8). In the absence of phage, SM02 counts increased by more than 2 log cycles. In contrast, phage-treated samples showed a dose-dependent reduction in bacterial counts. At MOI 0.1, the bacterial count decreased from 4.95 to 3.22 log_10_ CFU/mL. At MOI 1.0, the count decreased from 4.28 to 2.29 log_10_ CFU/mL, while at MOI 5.0, the count further decreased to 2.18 log_10_ CFU/mL by day 7.

### 3.10. Inhibition of S. marcescens in Soft Cheese

**Figure 9:**
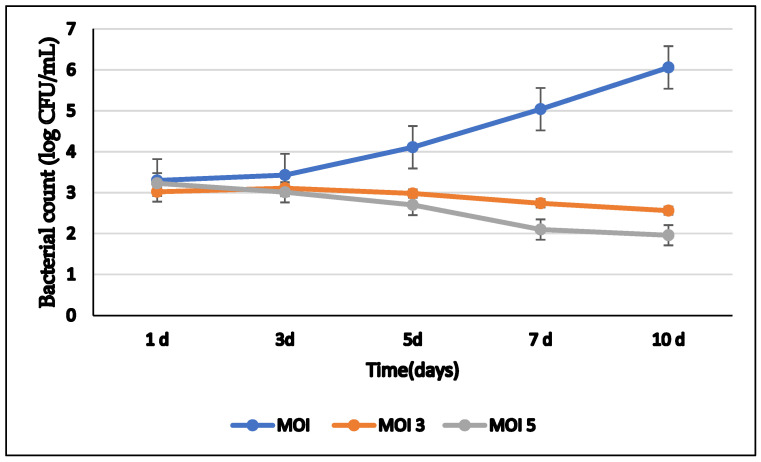
Effect of phage ØAS2 at different multiplicities of infection (MOIs) on *S. marcescens* SM02 in soft cheese during refrigerated storage for 10 days. Bacterial counts are expressed as log CFU/mL. Data are presented as mean ± standard deviation (SD) from three independent experiments (n = 3). Error bars represent SD.

In soft cheese stored under refrigerated conditions for 10 days, *S. marcescens* SM02 counts increased from 3.3 to 6.06 log_10_ CFU/mL in the absence of phage (Figure 9). In contrast, phage-treated samples exhibited a marked reduction in bacterial counts in a dose-dependent manner. At MOI 3.0, the count decreased from 3.02 to 2.56 log_10_ CFU/mL by day 10. At MOI 5.0, the count decreased to 2.1 log_10_ CFU/mL by day 7 and further to 1.96 log_10_ CFU/mL by day 10.

## 4. Discussion

The rapid emergence of multidrug-resistant *S. marcescens* poses a serious public health threat, particularly in healthcare and food processing environments [2,3]. In this study, we isolated and characterized a novel lytic phage (ØAS2) that is a member of the class *Caudoviricetes* that exhibits podovirus-like morphology, and evaluated its efficacy as a biocontrol agent against *S. marcescens* in milk and soft cheese.

The antibiotic susceptibility profile of SM02 revealed resistance to 50% of the tested antibiotics, including lincomycin, amikacin, erythromycin, amoxycillin, penicillin, and vancomycin, while showing sensitivity to fluoroquinolones and cefepime. These findings are consistent with previous reports of *S. marcescens* resistance patterns [29,30].

Phage ØAS2 formed clear plaques with a surrounding halo, indicative of depolymerase activity, which enhances its anti-biofilm potential [31,32]. Plaque halos were observed, which may suggest the presence of depolymerase activity, but this remains speculative without direct enzymatic assays or genomic evidence. TEM analysis revealed that phage ØAS2 exhibited a *podovirus*-like morphology consistent with members of the class *Caudoviricetes,* similar to previously described *Serratia* phages [33,34].

The relatively broad host range of ØAS2, including activity against *Klebsiella*, *E. coli*, *Salmonella*, and *Shigella*, is consistent with previously reported broad-spectrum *Serratia* phages [34,35].

The rapid adsorption rate (99.51% within 10 min) and short latent period (10 min) are advantageous for biocontrol applications, as they allow for rapid bacterial suppression [18]. The burst size of 115 PFU/cell is comparable to other *Serratia* phages [33,35].

ØAS2 demonstrated broad pH stability (4–11) and thermostability up to 60 °C, which are desirable traits for food applications, particularly in dairy processing where thermal treatments are common [18].

The biological properties of phage ØAS2 compare favorably with those reported for previously characterized lytic phages infecting *Serratia marcescens*. The broad host range observed in the present study, which extended beyond *S. marcescens* to include several Gram-negative bacteria such as *Escherichia coli*, *Salmonella Typhi*, *Shigella* spp., and *Klebsiella* spp., is consistent with reports of polyvalent *Serratia* phages capable of infecting multiple members of the *Enterobacteriaceae* [35]. Such a host spectrum may increase the practical utility of ØAS2 in complex food-processing environments where mixed bacterial populations frequently coexist. Moreover, the ability of ØAS2 to remain stable across a wide pH range (4–11) and at temperatures up to 60 °C further supports its suitability for food-related applications. Similar environmental tolerance has been reported for other food-associated bacteriophages and is considered an important prerequisite for successful biocontrol under industrial processing and storage conditions [36]. The combination of rapid adsorption, a short latent period, high burst size, and environmental stability suggests that ØAS2 possesses characteristics desirable for effective bacterial control. Collectively, these features indicate that phage ØAS2 may represent a promising candidate for incorporation into phage-based intervention strategies aimed at reducing contamination by multidrug-resistant and biofilm-forming *S. marcescens* in dairy production systems. Compared with previously reported *Serratia* phages, ØAS2 exhibited competitive biological characteristics, including a shorter latent period and comparable burst size. These properties further support its potential suitability for biocontrol applications in dairy environments [37].

The ability of ØAS2 to significantly reduce both planktonic growth and preformed biofilms of *S. marcescens* is particularly important, as biofilm formation represents a major challenge in the dairy industry [4,29]. The observed dose-dependent reduction in biofilm biomass, with the highest effect at MOI 5.0, is consistent with previous phage-mediated biofilm eradication studies [29,37]. In skim milk and soft cheese, ØAS2 effectively controlled SM02 contamination under refrigerated storage. These results align with previous studies demonstrating the efficacy of phages in dairy products against *Pseudomonas*, *Staphylococcus*, and *Listeria* [36,38,39]. The successful application of ØAS2 in soft cheese is particularly significant, as limited studies have explored *Serratia* phages in this matrix. These findings further support the growing evidence that bacteriophages represent effective and environmentally friendly alternatives for controlling multidrug-resistant bacteria in food systems.

## 5. Limitations

The present study has several limitations that should be acknowledged. First, the host range evaluation was performed using a relatively limited number of Serratia marcescens isolates, which may not fully represent the genetic and phenotypic diversity of this species. In addition, host susceptibility was assessed primarily through qualitative lytic activity assays, while efficiency of plating (EOP) analysis was not performed. Therefore, although phage ØAS2 demonstrated activity against multiple bacterial hosts, the relative efficiency of infection and replication across susceptible strains remains to be determined.

Second, whole-genome sequencing of phage ØAS2 was not conducted. Consequently, definitive taxonomic classification in accordance with current ICTV recommendations could not be established, and the presence of lysogeny-associated genes, virulence factors, antimicrobial resistance genes (ARGs), or other undesirable genetic elements could not be assessed. Future genomic investigations are required to confirm the taxonomy, safety profile, and suitability of ØAS2 for food-related biocontrol applications.

Third, the pH stability assay was conducted under a single temperature condition (4 °C), while thermal stability was evaluated using a relatively short exposure period of 10 min. Although the results demonstrated considerable environmental stability of the phage, additional studies investigating the combined effects of pH, temperature, and extended exposure periods are needed to better simulate practical food-processing and storage conditions.

Fourth, the antibacterial activity of ØAS2 was primarily evaluated using optical density measurements as an indirect indicator of bacterial growth inhibition. While this approach is widely used for preliminary assessment of phage–bacteria interactions, viable cell enumeration would provide a more direct and accurate measure of bacterial reduction and should be included in future studies.

Fifth, the antibiofilm activity of ØAS2 was assessed solely through crystal violet staining, which measures total biofilm biomass and does not distinguish between viable and non-viable cells. Therefore, the observed reductions reflect changes in biofilm biomass rather than confirmed bacterial killing within the biofilm structure. Future investigations should incorporate viable cell counts, live/dead staining, confocal laser scanning microscopy, or other complementary techniques to better characterize the antibiofilm efficacy of the phage.

Finally, phage persistence and long-term stability in dairy matrices were not directly evaluated, and potential effects of phage application on organoleptic properties, including taste, odor, texture, and appearance, were not investigated. In addition, spoilage-associated physicochemical parameters were not measured in the present study. Future research should address these aspects to further validate the practical applicability of phage ØAS2 for industrial-scale biocontrol and food preservation purposes.

## 6. Conclusions

In this study, a novel lytic bacteriophage (ØAS2) exhibiting *podovirus*-like morphology and belonging to the class *Caudoviricetes* was successfully isolated and characterized. ØAS2 exhibited a short latent period (10 min), a burst size of 115 PFU/cell, broad host range, high pH stability (4–11), and thermostability up to 60 °C. The phage effectively inhibited planktonic growth and eradicated preformed biofilms of *S. marcescens* in vitro at various MOIs. When applied to skim milk and fresh soft cheese under refrigerated storage, ØAS2 significantly reduced bacterial contamination. Collectively, the findings of this study demonstrate that phage ØAS2 is a promising biocontrol candidate capable of reducing *S. marcescens* populations and biofilms in dairy matrices. These results support the potential application of phage-based strategies for controlling multidrug-resistant *S. marcescens* in dairy production environments. Further studies are warranted to evaluate the impact of phage treatment on spoilage-associated parameters and product shelf life. Future studies should explore its application in combination with other phages or under different processing conditions to further enhance its utility in the dairy industry.

## Figures and Tables

**Table 1:** Antibiotic susceptibility of *S. marcescens* SM02 according to [15] CLSI M100 (2024) guidelines.

Antibiotic	Concentration	Inhibition Zone (Mean ± SD)	CLSI Interpretation
Lincomycin	10 µg	6.00 ± 0.00	NA
Amikacin	30 µg	6.00 ± 0.00	R
Erythromycin	15 µg	6.00 ± 0.00	NA
Kanamycin	30 µg	14.66 ± 0.57	I
Azithromycin	15 µg	23.00 ± 1.00	S
Moxifloxacin	5 µg	26.00 ± 1.00	S
Amoxicillin	10 µg	6.00 ± 0.00	R
Penicillin	10 units	6.00 ± 0.00	NA
Cefepime	30 µg	27.33 ± 0.58	S
Imipenem	10 µg	20.33 ± 0.57	S
Ofloxacin	5 µg	40.33 ± 0.57	S
Vancomycin	30 µg	6.00 ± 0.00	NA

S, susceptible; I, intermediate; R, resistant; NA, not applicable. Interpretations were assigned according to CLSI M100 (2024) criteria for *Enterobacterales* when available. For antimicrobial agents lacking CLSI interpretive criteria for *S. marcescens*, only inhibition zone diameters are reported and no susceptibility category was assigned.

**Table 2:** Host range of phage ØAS2.

Bacterial Strain	Reaction
*S. marcescens* SM02 PZ282064.1 (main host)	+
*Serratia marcescens* SM01 PZ273769.1	−
*Serratia marcescens* SM03	−
*Serratia marcescens* SM04	+
*Serratia marcescens* SM05	+
*E. coli* ATCC 25922	+
*E. coli* M30LC649234.1	−
*Salmonella typhi* 1	−
*Salmonella typhi* ATCC 14028	+
*Shigella* spp.	+
*Klebsiella oxytoca* KO37	+
*K. pneumoniae KP77* OK326738.1	+
*Klebsiella pneumoniae* KP98	−
*K. pneumoniae* KP75 OK326736.1	−
*K. pneumoniae* KP72 OK326732.1	−

(+): lytic activity; (−): no lytic activity. Quantitative plaque measurements were not available for all host strains; therefore, qualitative lysis results are presented.

## Data Availability

The original contributions presented in this study are included in the article. Further inquiries can be directed to the corresponding author.
